# Insights From Pre-Clinical and Clinical Studies on the Role of Innate Inflammation in Atherosclerosis Regression

**DOI:** 10.3389/fcvm.2018.00032

**Published:** 2018-05-11

**Authors:** Karishma Rahman, Edward A. Fisher

**Affiliations:** Department of Medicine, Division of Cardiology, New York University School of Medicine, New York, NY, United States

**Keywords:** innate immunity, macrophages, atherosclerosis progression, atherosclerosis regression, pre-clinical models, clinical trials as topic

## Abstract

Atherosclerosis, the underlying cause of coronary artery (CAD) and other cardiovascular diseases, is initiated by macrophage-mediated immune responses to lipoprotein and cholesterol accumulation in artery walls, which result in the formation of plaques. Unlike at other sites of inflammation, the immune response becomes maladaptive and inflammation fails to resolve. The most common treatment for reducing the risk from atherosclerosis is low density lipoprotein cholesterol (LDL-C) lowering. Studies have shown, however, that while significant lowering of LDL-C reduces the risk of heart attacks to some degree, there is still residual risk for the majority of the population. We and others have observed “residual inflammatory risk” of atherosclerosis after plasma cholesterol lowering in pre-clinical studies, and that this phenomenon is clinically relevant has been dramatically reinforced by the recent Canakinumab Anti-inflammatory Thrombosis Outcomes Study (CANTOS) trial. This review will summarize the role of the innate immune system, specifically macrophages, in atherosclerosis progression and regression, as well as the pre-clinical and clinical models that have provided significant insights into molecular pathways involved in the resolution of plaque inflammation and plaque regression. Partnered with clinical studies that can be envisioned in the post-CANTOS period, including progress in developing targeted plaque therapies, we expect that pre-clinical studies advancing on the path summarized in this review, already revealing key mechanisms, will continue to be essential contributors to achieve the goals of dampening plaque inflammation and inducing its resolution in order to maximize the therapeutic benefits of conventional risk factor modifications, such as LDL-C lowering.

## Introduction

Atherosclerosis, which underlies coronary artery disease (CAD), is characterized by a maladaptive immune response to lipoprotein and cholesterol accumulation in artery walls that results in the formation of plaques (also called lesions). The Pathobiological Determinants of Atherosclerosis in Youth study demonstrated that established coronary artery plaques begin their progression in childhood (1). Furthermore, a majority of patients likely have formation of advanced plaques even before physical symptoms of CAD, such as angina, manifest (2). Since plaques are established so early, efforts to reduce the morbidity and mortality of CAD requires inducing favorable changes to pre-existing clinical disease (3–6). Unfortunately, there is ample evidence that the conventional risk reduction therapies, heavily weighted towards reducing low density lipoprotein cholesterol (LDL-C) levels, leave a large amount of residual risk (7). This motivated our group and others to develop pre-clinical models of atherosclerosis regression and study them in molecular detail in order to identify potential clinical approaches and therapeutic targets to reduce the residual risk. In this review, we will summarize the evidence for the role of the innate immune system, focusing on plaque macrophages, in both atherosclerosis progression and regression, as the large body of work in the former has informed the design and interpretations of the latter. We should first note that we are aware of the participation of the adaptive immune system in atherosclerosis. As plaques advance, T and B-cells, in particular, make increasingly important contributions to the inflammatory state of plaques and to the local and systemic responses to antigenic material generated from modification of apolipoprotein B (APOB)-containing lipoproteins (LPs) and from tissue damage and cell necrosis. For this topic, we refer the reader to other expert reviews, such as (8–17).

## Macrophages in Atherosclerosis Progression

Atherosclerotic lesion development begins with the accumulation of cholesterol-rich APOB-LPs, which include very low density lipoprotein (VLDL) and LDL particles, in the subendothelial space (18, 19). Circulating monocytes derived from the bone marrow and spleen enter the subendothelial space of the arteries, with some of them differentiating to macrophages, which in turn, ingest retained lipoproteins in probably both their native and modified forms [e.g., oxidized LDL (OxLDL)], and become activated. Activation of macrophages (and endothelial cells) also leads to the secretion of chemoattractant molecules such as CCL2 (MCP-1) and CCL5 (RANTES) that lead to further recruitment of circulating monocytes.

Humans have two main subsets of circulating monocytes, Cluster of Differentiation 14^+^ (CD14^+^) CD16^−^ and CD14^low^CD16^+^, which correspond, respectively, to the lymphocyte antigen 6C (Ly6C)^high^ and Ly6C^low^ monocyte subsets in mice. These circulating monocytes, via CC-chemokine ligand 5 (CCL5) and CXC-chemokine ligand 1 (CXCL1), bind to endothelial cell glycosaminoglycans and P-selectin, as well as to vascular adhesion molecule 1 (VCAM1) and intracellular adhesion molecule 1 (ICAM1) binding via integrins very late antigen 4 (VLA4) and lymphocyte function-associated antigen 1 (LFA1), respectively. Together, these factors allow for monocyte adhesion to the activated endothelial cell layer (3, 20, 21).

The monocytes then transmigrate into the subendothelial space using surface receptors CC-chemokine receptor 2 (CCR2), CX3- chemokine receptor 1(CX3CR1), and CCR5, which bind to chemoattractant proteins released from the endothelial cells, as well as from existing macrophages in the plaque, such as CCL2, CX3CRL1, and CCL5, respectively (3, 20, 21). Importantly, it has been found that Ly6C^high^ and Ly6C^low^ monocytes differentially use these chemokine receptors for migration into plaques. Ly6C^high^ monocytes preferentially use CCR2 and CX3CR1, while Ly6C^low^ monocytes preferentially use CCR5 to enter plaques during plaque progression (22). Furthermore, the combined deficiency of these 3 receptor-ligand interactions led to a ~ 90% decrease in atherosclerosis burden (23), with the major fraction due to the loss of CCR2-CCL2 mediated monocyte migration into plaques (24, 25). Additionally, hypercholesteremia induces increased CCR2 expression in monocytes, leading to increased migration to CCL2, as well as Ly6C^high^ monocytosis (26–28). This suggests that Ly6C^high^ monocyte recruitment, mediated by CCR2 and CCL2, to the plaque is essential for atherosclerosis progression. Furthermore, loss of macrophage colony stimulating factor (M-CSF), which stimulates differentiation of monocytes to macrophages, led to almost complete loss of plaque development (29, 30).

Monocyte-derived macrophages ingest APOB-LPs, particularly LDL, through the LDL receptor (LDLR), beginning the formation of foam cells, a key step in the initiation and progression of atherosclerotic lesions (3, 20, 21). However, LDLR expression quickly decreases due to increased intracellular cholesterol levels (3, 21). Some of the remaining LDL becomes modified in a number of ways, including by oxidation (OxLDL) in the artery wall (31). After LDLR downregulation, macrophages then take up normal and modified LDL particles via pinocytosis and binding to scavenger receptors, most notably scavenger receptor A1 (SR-A1) and CD36, eventually becoming overwhelmed by the ingested lipids (3, 21, 32–34). As macrophages accumulate intracellular cholesterol, they upregulate molecules involved in cholesterol efflux pathways, such as ATP-binding cassette subfamily A member 1 (ABCA1) and ABCG1, via cholesterol precursors or derivatives that induce Liver X Receptor (LXR) activation, to induce the removal of intracellular cholesterol (3, 21). ABCA1 promotes efflux to lipid-poor apolipoprotein A1 (APOA1), which is a major component of high density lipoprotein (HDL) particles, while ABCG1 promotes efflux to mature lipid-rich HDL particles (35, 36). Despite this attempt at restoring homeostasis in cellular lipid content, plaques macrophages continue to accumulate cholesterol, which has many adverse effects, as will be described in a number of places in this review.

**Figure 1:**
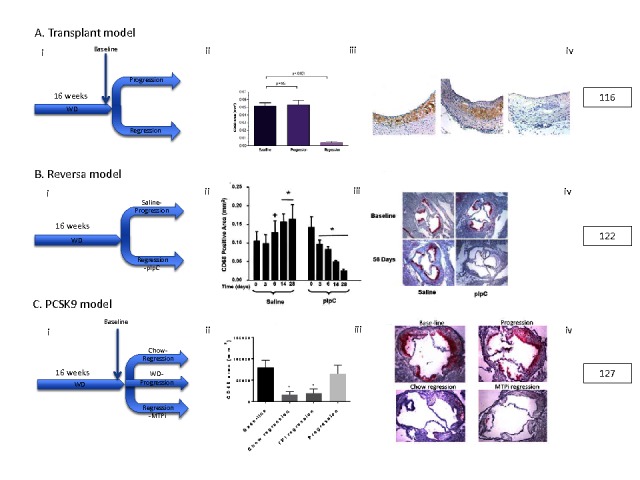
Representative Murine Plaque Regression Models. Selected models taken from those described in the text: **(A)** Transplant Model, **(B)** Reversa Model,and **(C)** PCSK9 model. For each model: (i) Schematic of experiment, (ii) Quantification of CD68^+^ (macrophage) area, *<0.05, (iii) representative CD68^+^ stained immunohistochemical sections, and (iv) original reference from which the images are modified from. Despite different methods to drastically lower circulating lipids,all of these models show a significant (*<0.05) reduction in CD68^+^ macrophage content in regression groups compared to respective baseline and/or progression groups.

Macrophages have multiple roles in inflammation, for example, by promoting it during infection or by resolving it during wound and tissue repair (37–39). One influential classification of macrophages that recognizes the wide range of macrophage inflammatory phenotypes- from causative to protective- is based on the work of Siamon Gordon and Alberto Mantovani and their colleagues, who broadly described M1 (classically activated) and M2 (alternatively activated) states (40, 41). Since the initial descriptions of the M1 and M2 states, there has been much research- and controversy- about the numbers of sub-types of macrophages not only within each category, but also along the spectrum between the categories [e.g., (39)]. Indeed, we have participated in a recent perspective on macrophage activation and polarization (42). For the purposes of this review, as summarized in Figure 1 of that perspective, our referring to M1 or M2 macrophages in mice corresponds to M[lipopolysaccharide (LPS)] or M[ Interleukin-4 (IL-4)], respectively, based on the markers we have documented in macrophages in progressing and regressing plaques (43, 44). Looking ahead, as advanced sequencing techniques, including but not limited to single cell RNA sequencing and CEL-Seq2 (45–47), become incorporated into atherosclerosis studies, the phenotyping of plaque macrophages will undoubtedly continue to be refined.

*In vitro*, macrophages are typically polarized towards M1 by incubation with LPS alone or LPS combined with interferon γ (IFNγ), which activates Toll-like receptor-4 (TLR-4) and its downstream effector, nuclear factor kappa-light-chain-enhancer of activated B cells (NF-κB). M2 polarization can be induced by interleukin-13 (IL-13) or, more commonly, IL-4, via activation of IL-4/IL-13 receptor and its downstream effector, signal transducer and activator of transcription 6 (STAT6) (40, 41, 48). M1 macrophages promote inflammation by highly expressing pro-inflammatory mediators, such as inducible nitric oxide synthase (iNOS), tumor necrosis factor-α (TNF-α), interleukin 1β (IL-1β), IL-6, and IL-12. M2 macrophages resolve inflammation by expressing anti-inflammatory mediators, such as transforming growth factor β (TGF-β), IL-1 receptor antagonist, and IL-10, which also induces M2 macrophage polarization and collagen production (49). In reality, macrophages span a range of phenotypes, but the M1/M2 scheme has nevertheless served as a convenient classification system. As advanced sequencing techniques (45–47) become incorporated into atherosclerosis studies, we will better be able to identify and understand the roles of the macrophage subsets during plaque progression and regression. For this review, we will focus on the M1/M2 paradigm as many of the studies we cite assess macrophage polarization within the simplified model.

There are multiple inflammatory stimuli that influence the macrophage polarization state in progressing plaques. One comes from the formation of cholesterol crystals (50, 51), which induces inflammasome activation and consequently IL-1β secretion. IL-1β is a potent inflammatory cytokine that promotes atherosclerosis progression as well as M1 polarization (15, 51–53). Cholesterol accumulation also increases plasma membrane cholesterol content, which sensitizes TLRs to their ligands, amplifying the inflammatory response (54, 55). As noted above, intracellular cholesterol accumulation activates LXR, which in addition to promoting cholesterol efflux, is also a repressor of NF-κB, leading to dampening of inflammation (3, 21, 49, 56, 57). However, the pro-inflammatory response and the associated M1 macrophage polarization predominates during plaque progression due to reinforcement by additional signals, such as TLR activation by pathogen-associated molecular patterns (PAMPS) and damage-associated molecular patterns (DAMPS; see below) (3, 21, 49). One PAMP is thought to be a lipid component of OxLDL that is recognized by TLR4 and leads to the production of TNF-α and IL-6 (58). OxLDL can also activate the TLR4-TLR6 heterodimer via CD36, leading to NF-κB activation and chemokine secretion that induces monocyte recruitment into plaques (3, 21, 59–61). Another inflammation-related mechanism involves T-cell infiltration into the plaque during progression. The infiltrating T-cells secrete inflammatory type 1 helper T cell (Th1) cytokines (IL-1, IL-6, TNF-α) that promote inflammation and M1 macrophage polarization (62–64), and in advanced plaques, inhibit smooth muscle cell collagen synthesis (65). Coupled with robust secretion by M1 macrophages of matrix metalloproteinases (MMPs) that degrade collagen, this results in thinning of the sub-endothelial fibrous cap, a protective and desirable component in human plaques, thought to prevent their rupture.

Furthermore, advanced atherosclerosis lesions are characterized by increased accumulation of dying/dead cells. There are a number of likely stimuli in progressing plaques that promote cell death by apoptosis, and by a related process, pyroptosis. These include the activation of ER stress by cholesterol, accumulation of reactive oxygen species, oxysterols and other modified lipids, and the activation of the inflammasome by multiple TLR ligands, TNF-α, and cholesterol crystals (3, 21, 50, 66–76). These deteriorating cells are normally cleared via efferocytosis, but as plaques advance, macrophages lose their efferocytosis capability. M2 macrophages are thought to have more efferocytosis capability than M1 macrophages, thus the predominance of M1 macrophages during plaque progression may also contribute to efferocytosis not being able to keep pace (49). This combination of increased accumulation of apoptotic cells and defective efferocytosis leads to secondary necrosis, which contributes to large lipid-filled necrotic cores and the release of more DAMPS (15, 77), which as noted above, are TLR ligands and stimulate inflammatory pathways. While secondary necrosis after inefficient efferocytosis of apoptotic or pyroptotic cells contributes to further inflammation and the formation of the necrotic core of plaques, recent studies have also highlighted the contributions to cell death of primary macrophage necrosis as a result of activation of the necroptosis pathway by TNF and OxLDL (78–81).

The combination of large necrotic cores and thinning of the fibrous cap destabilizes the plaque and increases risk of rupture and thrombus formation that precede heart attacks and strokes (2, 3, 15, 20, 21, 49, 82). In human plaques, M1 macrophages are localized to rupture prone regions, such as the necrotic core and plaque shoulder, while M2 macrophages are localized in the adventitia and farther away from the necrotic core (83, 84). This further solidifies the role of M1 macrophages in not only plaque progression, but also in the events that directly precede plaque rupture. This progression from the response to retained APOB-LPs to plaque rupture represents a maladaptive innate immune response. Normally, after the recruitment of monocytes to damaged tissues and the enrichment in M1 macrophages, there is eventual resolution of the inflammation and beneficial tissue remodeling by M2 macrophages (3, 15, 21, 28, 85). As will be presented below, the value of atherosclerosis regression models is to first illustrate that the failure to resolve inflammation during plaque progression can be overcome, and then to provide discovery platforms with which to accomplish this.

## Atherosclerosis Regression in Pre-Clinical Models

### Rabbits, Non-Human Primates, and Pigs

The history of plaque regression in rabbit, non-human primates, and pigs has been detailed extensively in our previous review (4). More briefly, evidence of plaque regression was observed as early as the 1920s, when it was observed that arterial lesions from cholesterol-fed rabbits that were switched to low-fat chow had increased fibrous content and reduced lipid content (86). In 1957, Friedman and colleagues performed one of the first prospective, interventional studies that showed plaque regression in cholesterol-fed rabbits that were injected with phosphatidylcholine (PC). Over the next two decades, similar plaque regression was observed in response to injections of dispersed phospholipids by multiple groups in experimental atherosclerosis models in rabbits (87–90) and baboons (91).

These findings were attributed to the ability of dispersed phospholipids to spontaneously form liposomes in aqueous solutions that can extract un-esterified cholesterol from membranes and cells, including plaque macrophages (90). These findings were further bolstered when Badimon and colleagues found that infusions of HDL, a known acceptor of cholesterol from macrophages, led to atherosclerosis regression in cholesterol fed-rabbits (92). Aikawa and Libby found that dietary lipid lowering led to plaque composition changes in rabbit atherosclerotic lesions that signaled increased plaque stability and reduced thrombotic potential. This included reduced tissue factor (TF) expression and activity (93, 94) along with decreased macrophage content, reduced expression and activity of MMPs, increased collagen accumulation, and increased smooth muscle cell area in the fibrous cap (94). Taken together, these studies provided strong evidence that dietary lipid lowering along with treatments to remove cholesterol from membranes and cells led to plaque regression in rabbit models of atherosclerosis (4, 90).

Further evidence of plaque regression was found in squirrel monkeys in 1968. A switch from an atherogenic to chow diet led to significant loss of lipid content compared to baseline lesions (95). This was further confirmed in rhesus monkeys (4, 96–100). A common theme in these studies was that lesions at varying stages of plaque progression (fatty streaks to more advanced lesions) all regressed due to dietary lipid depletion (96), consistent with findings in rabbit models of plaque regression.

Pigs have also been used for atherosclerosis regression research. It was found that after atherosclerosis was induced by a combination of arterial injury and a high cholesterol diet, there were favorable plaque composition changes including decreases in lipid content and necrotic core area after the animals were switched to a regular chow diet (101). In 1981, the same group published extensive histological analysis of plaques at different stages of regression. Interestingly, they reported that the advanced lesions showed changes compatible with a “healing process” characterized by the disappearance of foam cells, a significant decrease in necrotic areas, and increased replacement of necrotic debris by fibrous tissue and calcified areas (102). Furthermore, they reported that early in the regression process, the decrease in foam cells and necrotic areas was accompanied by an increase in non-foam cell macrophages (102). This led them to hypothesize that the disappearance of the necrotic core areas occurred because the necrotic debris was removed by newly recruited, functioning, healthy macrophages (102).

### Mice

Mice have naturally low LDL-C levels, with most of their plasma cholesterol carried by HDL, and it is thought that this plasma lipoprotein profile underlies their natural resistance to atherosclerosis (4). The use of murine models in atherosclerosis was catalyzed by the development of the apolipoprotein E (APOE) knockout mouse by the Breslow (103) and the Maeda (104) laboratories, and the LDL receptor (LDLR) knockout mouse by the Brown and Goldstein laboratory (105) in the early 1990s. In mice, APOE is the major ligand for the LDL receptor, even for APOB-containing lipoproteins. Thus, both models eliminate pathways for efficient lipoprotein cholesterol clearance by removing either a ligand (*Apoe*^−/−^) or a receptor (*Ldlr*^−/−^), and result in above normal plasma levels of cholesterol carried by APOB-lipoproteins. The circulating plasma levels of cholesterol can be further increased by feeding the mice a high fat and high cholesterol diet (“western diet”, WD), which accelerates the development of atherosclerotic plaques (4).

With mouse atherosclerosis progression models in place, we and others focused on adapting them for studies of regression. Similar to the rabbit, non-human primate, and pig studies, plaque regression was induced by significant lowering of circulating lipid levels, especially LDL-C, or by elevating HDL particles either genetically or by infusion (4, 49, 106). Significant LDL-C lowering could not be accomplished, however, by statin treatment, given the absence of either the ligand for the LDL receptor or the receptor itself. One approach to lipid lowering involved the hepatic overexpression of *Apoe* in *Apoe*^−/−^ mice (107–111) and *Ldlr* in *Ldlr*^−/−^ mice (105, 112–115) using adenoviral-mediated gene transfer. Both approaches led to the normalization of atherogenic lipid profiles, and favorable changes in plaque size, composition, or macrophage content (4, 105, 107–115). However, in many of these models, the regression of early fatty streaks was much more pronounced than in advanced lesions. While fatty streaks reduced in lesion size by a factor of 10 compared to baseline, advanced lesions usually had smaller reductions in size by <20% (110, 116). This difference in plaque regression may have been due to the transient nature of the adenoviral-mediated lipid lowering with the early vectors, in part because of an immune response against virally transduced cells. Thus, models were needed in which lower lipid levels could be sustained indefinitely so that the regression of lesions of any complexity could be studied (116).

Towards this goal, the Fisher lab developed a plaque regression model that involved transplanting an atherosclerotic thoracic arch (116) or aortic arch segment (117) from a hyperlipidemic donor (*Apoe*^−/−^ fed a WD) into a normolipidemic recipient (wild type (WT) fed a chow diet). This rapid environmental change in circulating lipoprotein/lipid levels induced plaque regression over a surprisingly short time (starting at 3 days) (43, 116–120). The regression group plaques showed significantly decreased lesion, macrophage, and lipid areas compared to their baseline counterparts.

Another regression model developed by our lab (in collaboration with Dr. Stephen Young, UCLA) is the Reversa mouse (*Ldlr^−/−^ Apob*^ 100/100^* Mttp*^ fl/fl^*Mx1Cre*^+/+^) (121, 122), where after plaque progression occurs while the mice are on WD, polyinosinic-polycytidylic (pIpC) injection induces the Mx1-Cre gene leading to the inactivation of the microsomal triglyceride transfer protein (MTTP) gene. MTTP inactivation inhibits APOB-lipoprotein particle secretion from the liver. The resulting decrease of circulating cholesterol-rich VLDL and LDL induced plaque regression characterized by reduced macrophage content, reduced lipid area, and increased collagen content (122–124). Similar plaque regression was seen with treatment with a MTTP inhibitor in WD fed *Ldlr*^−/−^ mice (125).

A common denominator in the above models of plaque regression was the requirement of an intervention to reduce lipid levels considerably below what could be achieved by switching from WD to chow alone. However, Nagareddy and colleagues have shown that with longer periods of moderate lipid lowering induced by switching the WD to chow after plaques have been established in *Ldlr*^−/−^ mice, regression can occur (126).

Space does not permit their full descriptions, but a rapid expansion of the number of non-surgical models to study atherosclerosis regression is expected based on the combined use of PCSK9-adenovectors to make mice LDLR-deficient, followed by antisense oligonucleotides (ASOs) against APOB or MTTP to lower APOB-lipoproteins (127, 128). In addition, there are still other regression models that are based on non-LDL lowering strategies, such as raising HDL particles either transgenetically, by infusion of HDL, and treatment with anti-miR-33 (43, 106, 123, 129, 130). As will be presented later, there has been remarkable consistency in the changes in the macrophage properties in the plaques, independent of the mode of  regression.

In summary, plaque regression has been observed in multiple pre-clinical models (see Figure 1 for representative results from key models described above). We will next review the evidence of plaque regression in humans.

## Atherosclerosis Regression in Humans

One of the first reports of possible regression of established human atherosclerotic lesions was seen in autopsy studies by Aschoff in 1924 when he observed that there was reduced atherosclerosis severity during the famine Germany suffered after World War I (131). These findings were confirmed in 1946 by Vartianen and Kanerva (132), in 1947 by Wilens (133), and in 1951 by Wanscher and colleagues (134) in diverse populations that had suffered from severe food restrictions, chronic wasting diseases, and cancer-induced cachexia (135), suggesting that atherosclerosis severity could decrease after lesions were established.

In 1966, Ost and Stenson performed one of the first prospective, interventional studies that demonstrated plaque regression in humans. They found that 10% of patients treated with niacin, which lowers triglycerides, raises HDL-C, and lowers LDL-C, showed improved femoral angiograms as measured by reduction in flow-limiting stenosis (4, 136). Brown and colleagues summarized larger trials from 1984 to 1993 that examined the relationship between lipid therapies and plaque regression, as measured by angiographic evidence of improved arterial flow or changes in plaque size compared to their baseline angiograms (137). They found that while the absolute decreases in arterial narrowing after lipid therapies were statistically significant, they were remarkably small, especially compared to the reduction in adverse clinical events (137). This discrepancy between absolute changes in angiographic arterial narrowing and reduction in coronary events was termed the “angiographic paradox” (4). The paradox was at least in part clarified when it was found that vulnerable plaques prone to the thrombotic events preceding heart attack and stroke cause less than 50% stenosis compared to stable, but more occlusive, lesions (4, 137, 138). An interpretation we favor, based on the pre-clinical models, for example a few reviewed in (122, 124, 125), is that lipid lowering most likely led to remodeling and stabilization of the smaller, vulnerable plaques, some perhaps not even readily visible on the angiograms (137). This suggests that lipid lowering interventions can induce beneficial changes in plaques (via compositional changes that lead to stabilization of vulnerable plaques and significant reduction in coronary events), that imaging methods then (and, for that matter, now) are inadequate to easily detect.

Later trials switched from angiographic analysis to intravascular ultrasonography (IVUS) because of being able to measure arterial wall thickness (assumed to be equivalent to plaque volume) in addition to vascular lumen size (4). For example, Nissen and colleagues reported two major prospective trials that observed patients with CAD who were treated with high dose statins using IVUS: (1) the Reversal of Atherosclerosis with Aggressive Lipid Lowering (REVERSAL) trial (139), and, (2) A Study to Evaluate the Effect of Rosuvastatin on Intravascular Ultrasound-Derived Coronary Atheroma Burden (ASTEROID) (140). In the REVERSAL study, they found that LDL-C reduction greater than 50 percent in patients treated with atorvastatin for 18 months was associated with decreases in plaque volume of 0.4 percent (139). In the ASTEROID trial, LDL-C dropped from 130.4 mg/dl to an average of 60.8 mg/dl with rosuvastatin treatment for 24 months, and this was associated with a 0.98 percent decrease in plaque volume (140). The Study of Coronary Atheroma by Intravascular Ultrasound: Effect of Rosuvastatin versus Atorvastatin (SATURN) showed similar findings using IVUS, where plaque volume decreased by 0.99 percent in the atorvastatin treatment group and by 1.22 percent in the rosuvastatin treatment group after 24 months of LDL-C lowering treatment (141).

More recently, Nicholls and colleagues assessed the efficacy of proprotein convertase subtilisin kexin type 9 (PCSK9) antibodies to lower circulating LDL-C in the Global Assessment of Plaque Regression With a PCSK9 Antibody as Measured by Intravascular Ultrasound (GLAGOV) trial (142). They found that the group treated with PCSK9 antibody had significantly lower LDL-C levels compared to controls (36.6 mg/dL compared to 93.0 mg/dL after 76 weeks of treatment) and a 0.95 percent decrease in plaque volume compared to baseline (142). These studies provide consistent evidence that extensive lipid lowering can lead to plaque regression as measured by decreases in plaque volume. The modest quantitative changes in plaque volume, however, recall the surprising results of Brown and colleagues, which suggested that changes in plaque composition might pre-dominate over changes in plaque size after lipid-based interventions. As implied above, confirmation of this hypothesis will require more sensitive, preferably non-invasive, imaging methods as reviewed in (143).

Another therapeutic intervention that has been recently studied using IVUS was the infusion of HDL-like complexes into patients with acute coronary syndromes (ACS). Nissen and colleagues used APOA1-Milano. This form was originally identified in 40 carriers in northern Italy who had very low levels of HDL-C, but less CAD risk than expected (144, 145), suggesting increased HDL functionality, for example by removal of more cholesterol from plaque foam cells. They found that the group treated with APOA1-Milano infusions for 5 weeks had a 1.06 percent decrease in plaque volume compared with baseline volume (144). Interestingly, they found that increasing the dose from 15 to 45 mg/kg did not improve the results (144). In a subsequent study, Tardif and colleagues used reconstituted HDL using wild-type APOA1, and found that patients treated for 4 weeks had a 3.4 percent decrease in plaque volume compared to baseline (146).

The impact of these HDL infusion results on clinical thinking, however, has been limited not only by their small size, but also by a number of recent studies in which elevations in HDL-C are not necessarily linked to reductions in major adverse cardiovascular events. These studies include mendelian-randomization genetic (147–149), as well as HDL-C raising interventions with niacin (150, 151) or cholesteryl ester transfer protein (CETP) inhibitors (152–154). These results have highlighted the differentiation of the level of HDL-C from the function of HDL particles, with the latter being considered to be more strongly associated with risk protection (155). Thus, the promise of the HDL infusion and the disappointment in the above cited mendelian randomization and intervention studies may reflect the different functional capabilities of the HDL particles depending on the method of increasing their levels in circulation (4, 155, 156). In particular, the results from the HDL infusion studies may reflect the ability of increased numbers of *functional* HDL particles to favorably remodel plaques, as we and others have observed in mouse (43, 106, 157) and rabbit models (92, 158).

In summary, observational studies going back to 1924 and in more recent intervention trials support the regressibility of atherosclerotic plaques in humans. In the intervention studies, plaque regression is typically small (<1%) with aggressive lipid reductions, yet where outcome data are available, this is associated with significantly fewer events, suggesting that plaque compositional changes, not detectable by IVUS or angiography (which predominately measure wall thickness or luminal stenosis, respectively), may be a major contributing factor. Imaging modalities to detect changes in plaque composition is a very active field (e.g., 131, 147–150). While there have been advances (such as the use of optical coherence tomography, near IR spectroscopy, and NMR) there are still limitations because of either invasiveness or limited applicability to coronary sites. Undoubtedly, further progress will be made so that coronary artery compositional changes will become more common outcomes of clinical studies. Even then, given the limited mechanistic information possible to glean from clinical studies, for the understanding of how extensive lipid lowering or other interventions can lead to plaque regression and compositional changes, as well as the identification of the molecular pathways within the plaques that may be therapeutic targets, the pre-clinical models described earlier are invaluable. These studies have led to a number of insights, particularly in regard to plaque macrophage biology, which will be reviewed next.

## Macrophages in Atherosclerosis Regression

In regressing atherosclerotic plaques, we have observed that there is reduced expression of classical inflammatory genes characteristic of M1 macrophages, such as CCL2, TNFα, and iNOS. This reduction coincides with increased expression of genes encoding markers of alternatively activated, anti-inflammatory, tissue-remodeling M2 macrophages, such as Arg1, mannose receptor (MR/CD206), CD163, C lectin receptor, and IL-10 in CD68^+^ cells (43, 118, 122, 124, 125). This intriguing finding is consistent with the studies in mouse models that have shown that loss of nuclear hormone receptor 77 (NUR77) or Krüppel-like factor 4 (KLF4), two factors associated with lower macrophage inflammation (i.e., a more “M2-like” phenotype) leads to more plaque inflammation and atherosclerosis progression (159–163). Additionally, the induction of IL-4 or IL-13 mediated M2 macrophage polarization also favorably changes plaque composition to a less inflammatory state and reduces atherosclerosis progression (3, 21, 164, 165). Thus, the enrichment in M2 macrophages is a signature of regressing plaques suggesting that the healthier microenvironment not only reduces the signals for macrophage activation, but also provides signals for M2 polarization. This results in reduced inflammation and favorable tissue remodeling (3), consistent with the results from altering the M1/M2 balance in progression.

We were interested in the source of the M2 macrophages in regressing plaques. We had previously observed that despite the rapid and significant lipid lowering we often used to induce regression, there was still ongoing recruitment of monocytes to the plaques (166), suggesting these new cells may be the precursors. Another possibility is that these M2 macrophages resulted from proliferation and polarization of existing macrophages already in the plaque (167, 168) that originated from circulating monocytes recruited during progression or from tissue resident macrophages of embryonic origin (yolk sac-derived) and maintained through self-renewal (169, 170). We did not find evidence of much proliferation of macrophages in regressing plaques [e.g., (44)], which focused our attention on the role of newly recruited monocytes.

Conventionally, it is thought that Ly6C^high^ monocytes are the precursors of M1 macrophages, while Ly6C^low^ monocytes are the precursors of M2 macrophage (22, 23, 27, 37). Using normolipidemic mice deficient in either CCR2 or CCR5, which are selectively used to recruit Ly6C^high^ or Ly6C^low^ monocytes, respectively into atherosclerotic plaques (22), we conclusively showed that M2 macrophages in regressing plaques were surprisingly derived from cells recruited from the Ly6C^high^ circulating subset after lipid lowering (44). That the enrichment in M2 macrophages was not just associated with, but was required for, regression was demonstrated in experiments using normolipidemic mice deficient in STAT6, which as noted above, is a required factor for canonical (i.e., IL-4 or IL13-induced) M2 polarization. As will also be noted in the next section, these results point to the limitations of lipid lowering alone to achieve atherosclerosis regression, as when this was achieved without being able to enrich in M2 macrophages, regression was impaired. The results also stimulated us to seek the nature of the signals that were responsible for the polarization of the newly recruited monocytes.

## Inflammation Resolution as a Therapeutic Target and Its Clinical Relevance

Besides the pre-clinical findings just reviewed, there is also ample evidence from the clinical literature to argue that new directions are clearly needed to augment the traditional approach of lowering LDL-C by statins in order to achieve atherosclerosis regression and cardiovascular risk reduction. This point was driven home by a summary of the results of 6 “mega-trials” of statins- the Scandinavian Simvastatin Survival Study (4S), the Cholesterol And Recurrent Events (CARE) study, the Air Force Coronary Atherosclerosis Prevention Study/Texas Coronary Atherosclerosis Prevention Study (AFCAPS/TexCAPS), the Long-term Intervention with Pravastatin in Ischemic Disease study (LIPID) study, and the Heart Protection Study (HPS). While all showed reductions in coronary heart disease events, two thirds of patients still experienced events while on statin treatment, clearly demonstrating that LDL-C lowering alone does not lead to optimal therapeutic benefit (7).

The recent Canakinumab Anti-inflammatory Thrombosis Outcomes Study (CANTOS) trial (171) has brought to clinical focus what our pre-clinical studies have shown, namely that the benefits of LDL-C lowering are enhanced when inflammation is reduced. In this 2 year study, patients at high risk of CAD were treated with statins to lower LDL-C to 82 mg/dL, with half also receiving an antibody to the potent inflammatory mediator IL-1β. Indeed, there were decreased events in those in the antibody group, which demonstrated the clinical importance of the “residual risk of inflammation” that persisted after LDL-C lowering in some patients, particularly those whose levels of CRP, considered to be a biomarker of inflammation, were ≥2 mg/dL (171, 172). The CANTOS results have antecedents in the Pravastatin or Atorvastatin Evaluation and Infection Therapy–Thrombolysis in Myocardial Infarction 22 (PROVE IT–TIMI 22) study (172). After intensive statin therapy, Ridker and colleagues found there were 4 groups of patients with differential risk of having a recurrent heart attack – (1) LDL ≥70 mg/dL, CRP ≥2 mg/L (highest risk), (2) LDL ≥70 mg/dL, CRP <2 mg/L, (3) LDL <70 mg/dL, CRP ≥2 mg/L, and (4) LDL <70 mg/dL, CRP <2 mg/L (lowest risk). Intriguingly, they found that patients with LDL ≥70 mg/dL, CRP <2 mg/L or LDL <70 mg/dL, CRP ≥2 mg/L had similar risk of recurrent risk, which suggested that heightened inflammation impeded the benefits of LDL-C lowering (172), consistent with the CANTOS results and our pre-clinical findings (44, 171).

Given the direct evidence it provides in humans, the CANTOS trial has catapulted the interest in clinical approaches to reducing plaque inflammation and promoting its resolution, whose benefits have heretofore been heavily based in pre-clinical settings. Potential therapies, in addition to enhancing STAT6-induced M2 macrophage polarization, could include using plaque targeted therapies to increase lipid-derived (e.g., resolvins) and protein mediators (e.g., cytokines) to promote resolution, by blocking inflammatory cell influx, promoting their egress, clearing pathogens and cellular debris, reducing inflammatory cytokines, increasing the clearance of dying macrophages by efferocytosis, and repairing tissue damage by creating an environment that promotes tissue remodeling M2 macrophage polarization (173, 174). In parallel developments, nano-vehicles to direct some of these therapies directly to plaques are being intensely pursued. These will allow not only more potent attacks on inflammation, but have the potential to avoid systemic adverse effects, such as the increased fatal infections observed with systemic IL-1β antagonism in the CANTOS trial.

## Concluding Remarks

**Figure 2:**
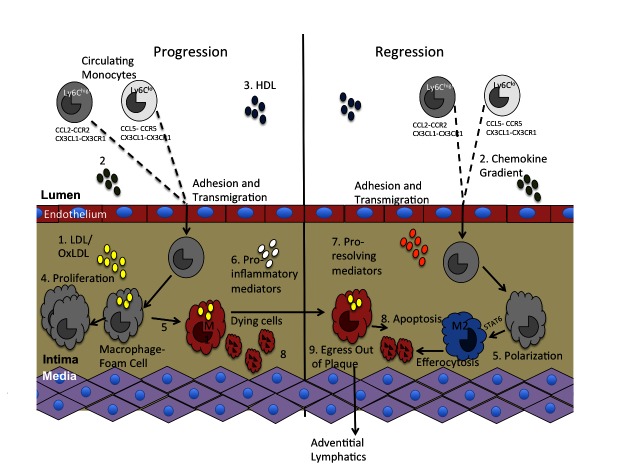
Key molecules involved in innate immunity and atherosclerosis pathogenesis. There are multiple molecules and pathways involved in the progression of atherosclerosis that have also been implicated in atherosclerosis regression. They include: 1. LDL/oxLDL, 2. Chemokines that induce monocyte recruitment into the plaque, 3. HDL, 4. Proliferation, 5. Polarization, 6. Pro-inflammatory mediators, 7. Pro-resolving mediators, 8. Apoptosis and efferocytosis, and 9. Macrophage egress out of the plaque.

It has been considered for some time [reviewed in, e.g., (15, 18, 175)] that atherosclerosis plaque progression, like many inflammatory processes, begins as a typical defense mechanism against a threat, in this case retained APOB-lipoproteins. For the reasons discussed above, there are a number of unfortunate events, such as the relentless entry of lipoproteins (which in addition to the lipids they bring, give rise to PAMPS), and the ensuing tissue damage, which produces DAMPS. These phenomena both amplify the innate immune responses and extend the mayhem to involve adaptive immunity. Thus, the normally homeostatic resolution of inflammation does not occur. As we have reviewed, progress in understanding the immunology of atherosclerosis based on pre-clinical models of progression and regression, coupled with advances in clinical investigations, puts the field well on its way towards the goal of achieving this homeostasis, which will result in improved primary and secondary prevention strategies and a reduction in atherosclerotic cardiovascular disease. In Figure 2 and Table 1, we have summarized some of the pathways and factors implicated in potentially limiting, or even better, resolving plaque inflammation.

**Table 1:** Key molecules and therapeutic potential for resolving innate immune inflammation during atherosclerosis progression and regression.

	Examples of Molecules	Examples of Therapeutics or Potential Approaches	Effect on Plaque Progression	Effect on Plaque Regression
LDL/oxLDL		Statins, PCSK9 Inhibitors, antibodies to oxidized lipids, vaccination to oxLDL	Retarded progression in humans	Lowering LDL leads to some regression in humans and more in animal models. Antibodies to oxLDL retard progression in mice; not tested in regression
Chemokine Gradient	CCL2/MCP-1, CCL5, CX3CRL1	CCR2 Inhibitors	Inhibiting chemokine function impairs progression in mice	Inhibiting chemokine function (CCR2) impairs regression in mice
HDL	ApoA1, ABCA1, CETP	HDL Infusions (ApoA1-Milano, ApoA1- WT), Niacin, CETP Inhibitors; anti-miR33	Increasing number of functional HDL particles (HDL-P) impairs progression in mice; limited human studies agree; raising HDL-C has not been effective	Increasing HDL-P via infusions leads to some regression in humans and significant regression in mice; anti-miR33 treatment leads to significant regression in mice; being tested in non-human primates
Pro-inflammatory mediators	Cytokines, e.g., IL-1β, IL-6, IL-12, TNF-α	Canakinumab (IL-1β Inhibitor)	Lowering inflammatory cytokines impairs progression in humans and mice	Not tested
	Leuokotrienes	5-lipoxygenase activating protein inhibitor	Reducing leukotrienes impairs progression in mice; polymorphisms in the gene associated with atherosclerosis in human genetic studies	Not tested
Pro-resolving mediators	Cytokines includiing IL-4, IL-10, IL-13	The cytokines themselves delivered systemically or as part of targeted nanoparticles	Timing and context matter but in general decreasing anti-inflammatory cytokines promotes, and increasing them retards, progression in mice	STAT6 (downstream of IL-4 and IL-13) required for regression in mice
	Lipoxins, resolvins, maresins, protectins	Treatment with annexin 1, resolvin D1, resolvin D2, maresin 1, and resolvin E1 systemically or as part of targeted nanoparticles	Increasing pro-resolving mediators impairs progression in mice	Not tested
Apoptosis/efferocytosis		Stimulators of efferocytosis, such as LXR agonists delivered as targeted nanomedicines given hepatic toxicity when given systemically	Early apoptosis and active efferocytosis can retard plaque progression but apoptosis and failed efferocytosis at advanced stages promote plaque progression by contributing to necrotic core formation in mice	Not tested
Emigration/Egress Out of Plaque	Netrin 1, CCR7	Delivery of siRNA or anti-sense oligonucleotides to netrin 1 in targeted nanoparticles	Netrins inhibit emigration of macrophages and promote progression in mice	Blocking CCR7 leads to impaired emigration of macrophages and impairs regression in mice

## Author Contributions

KR and EF wrote and edited the manuscript, and also created all figures.

## Conflict of Interest Statement

The authors declare that the research was conducted in the absence of any commercial or financial relationships that could be construed as a potential conflict of interest.
